# Case report: Exploring autosomal recessive woolly hair: genetic and scanning electron microscopic perspectives on a Japanese patient

**DOI:** 10.3389/fmed.2024.1374222

**Published:** 2024-05-16

**Authors:** Satoko Minakawa, Yasushi Matsuzaki, Toshihide Higashino, Tamio Suzuki, Hirofumi Tomita, Eijiro Akasaka, Daisuke Sawamura

**Affiliations:** ^1^Department of Dermatology, Hirosaki University Graduate School of Medicine, Hirosaki, Japan; ^2^Department of Clinical Laboratory, Hirosaki University Graduate School of Medicine, Hirosaki, Japan; ^3^Department of Human Genetics, Graduate School of Medicine, The University of Tokyo, Tokyo, Japan; ^4^Department of Dermatology, Faculty of Medicine, Yamagata University, Yamagata, Japan

**Keywords:** hypotrichosis, lipase H, *LIPH*, woolly hair, lysophosphatidic acid receptor 6

## Abstract

Woolly hair (WH) is a hair shaft anomaly characterized by tightly curled hair that typically stops growing at a few inches. Autosomal recessive WH (ARWH; OMIM no. 278150/604379/616760) has been reported to be caused by variants in genes coding lysophosphatidic acid receptor 6 (*LPAR6*), lipase H (*LIPH*), or keratin 25 (*KRT25*). In this study, we conducted a scanning electron microscopic (SEM) examination of the hair of a 3-year-old Japanese ARWH patient. The SEM revealed that her affected hair had an irregular and rough cuticle compared to her mother’s hair. Many irregular small projections and longitudinal grooves were seen on the surface of the patient’s hair shaft, and some free margins of the hair cortex were raised or serrated. Her hairs were oval-shaped on the cross-section. Mutation analysis revealed a homozygous pathogenic variant (c.736 T > A; Cys246Ser) in exon 6 in *LIPH*. In our clinic, we identified three additional cases with the homozygous Cys246Ser variant and one case with compound heterozygous variants in *LIPH*: Cys246Ser and c.671C > G (Pro224Arg). Consequently, genetic analyses, including genotype–phenotype correlation involving rare *LIPH* variants, have become more crucial in the Japanese population.

## Introduction

Woolly hair (WH) is a hair shaft anomaly characterized by tightly curled hair that typically stops growing at a few inches (1). Autosomal recessive WH (ARWH; OMIM no. 278150/604379/616760) has been reported to be caused by variants in genes coding lysophosphatidic acid receptor 6 (*LPAR6*), lipase H (*LIPH*), or keratin 25 (*KRT25*) (2–4).

## Case report

**Figure 1:**
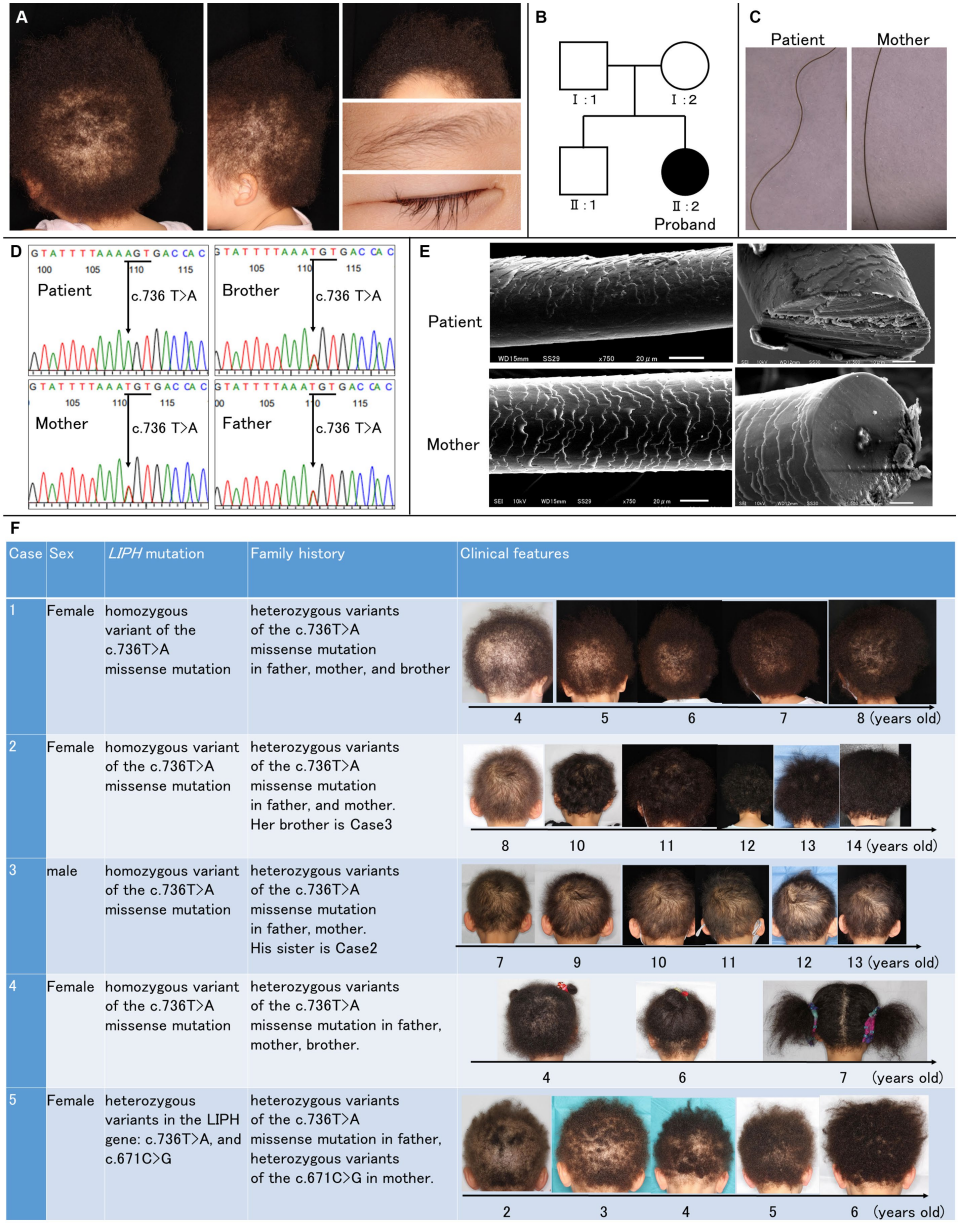
Clinical and genetic features of the patient. **(A)** Clinical manifestations of the patient included short and tightly curled woolly hair on the scalp, with noticeable scalp hair sparsity. Facial hair appeared normal. **(B)** Pedigree of the patient’s family, illustrating the autosomal-recessive inheritance pattern of woolly hair. **(C)** Trichoscopy images show undulated light brown hair in the patient compared to the mother. **(D)** Genetic analysis via direct sequencing identified a homozygous pathogenic variant (c.736 T > A; Cys246Ser) in exon 6 of *LIPH*. The Cys246Ser variant is segregated within the family. **(E)** Scanning electron microscopic images reveal irregular and rough cuticle structures in the patient’s affected hair, with oval-shaped cross-sections. **(F)** Clinical features of five cases. Cases 1–4 have the homozygous p.Cys246Ser variant. Case 5 has compound heterozygous variants: p.Cys246Ser and c.671C > G (p.Pro224Arg).

A 3-year-old Japanese girl has short and tightly curled scalp hair since birth, with sparse scalp hair evident (Figure 1A). Facial and body hair appear normal, albeit lighter in color compared to her mother’s hair. No signs of hormonal abnormalities, such as hirsutism, were observed, and routine laboratory findings were within the normal range.

Two years before visiting our clinic, she was diagnosed with atopic dermatitis. Her father and brother also had atopic dermatitis, and her brother had asthma. There was no family history of hair disorders (Figure 1B). The patient had no history of systemic diseases, trauma, or exposure to radiation or medications.

Under the trichoscopy, the hair of the patient exhibited undulation and was of a lighter brown shade than her mother’s hair. Her hair was thinner, and the thickness was uniform (Figure 1C). The direct sequencing of the coding exons in *LIPH* (NM_139248.3) revealed a homozygous pathogenic variant (rs201249971;c.736 T > A; p.Cys246Ser) in exon 6 (Figure 1D), and we diagnosed the patient as ARWH with the genetic and clinical features.

The scanning electron microscopic (SEM) examination revealed that her affected hair had an irregular and rough cuticle compared to her mother’s hair (Figure 1E). Many irregular small projections and longitudinal grooves were seen on the surface of the patient’s hair shaft, and some free margins of the hair cortex were raised or serrated. Her hair has shown gradual growth (Figure 1F; case 1).

The oval-shaped cross-section of the hair is considered to show signs of fragility in the outer cortex and a reduction of the medulla. The alteration in hair development aligns with the severe damage to the medulla observed via SEM.

In the Japanese population, most ARWH patients carry at least one of the two prevalent founder mutations in *LIPH*, p.Cys246Ser (minor allele frequency [MAF] in Japanese population (5)= 1.3%) and c.742C > A (rs201868115;p.His248Asn; MAF in Japanese population (5)= 0.095%) (6), while other variants have been occasionally identified. In our clinic, we confirmed that additional cases 2–4 have the homozygous p.Cys246Ser variant, and case 5 has compound heterozygous variants: p.Cys246Ser and c.671C > G (rs1453368208; p.Pro224Arg) through segregation analysis (Figure 1F). The p.Pro224Arg variant is absent in gnomAD (7) but identified as a rare variant with an MAF of approximately 0.01% in the Japanese population via TogoVar (5). Additionally, *in silico* prediction analyses yielded scores of 0.9179 for AlphaMissence (8), 32 for CADDv1.7 (9), 0 for SIFT (10), and 0.998 for PolyPhen-2, (11) indicating a high likelihood of a significant impact on protein function. To the best of our knowledge, p.Pro224Arg is detected as a causative variant for the first time.

Given the presence of two definitive founder mutations, rare variants in *LIPH* also serve as significant supplementary causative factors for ARWH within the Japanese population, particularly in the context of compound heterozygous variants. This suggests that Japanese individuals face a heightened risk of developing ARWH phenotypes specific to rare *LIPH* variants compared to other populations. Consequently, conducting genetic analyses, including genotype–phenotype correlations involving deep phenotyping of Japanese ARWH patients with rare *LIPH* variants, will prove beneficial in elucidating molecular features of *LIPH* and more intricate pathogenic mechanisms.

## Data availability statement

The datasets presented in this study can be found in online repositories. The names of the repository/repositories and accession number(s) can be found at: https://www.ncbi.nlm.nih.gov/snp/, rs201249971, rs201868115, and rs1453368208.

## Ethics statement

The studies involving humans were approved by approval of the research protocol: the protocol for this research project has been approved by a suitably constituted Ethics Committee of the institution and it conforms to the provisions of the Declaration of Helsinki. The study protocol was approved by the Committee of Medical Ethics of Hirosaki University Graduate School of Medicine. Approval No. 2020-146-10. The studies were conducted in accordance with the local legislation and institutional requirements. Written informed consent for participation in this study was provided by the participants’ legal guardians/next of kin. Written informed consent was obtained from the individual(s), and minor(s)’ legal guardian/next of kin, for the publication of any potentially identifiable images or data included in this article. Written informed consent was obtained from the participant/patient(s) for the publication of this case report.

## Author contributions

SM: Writing – original draft, Writing – review & editing. YM: Writing – review & editing. TH: Writing – review & editing. TS: Writing – review & editing. HT: Supervision, Writing – review & editing. EA: Visualization, Writing – review & editing. DS: Funding acquisition, Writing – review & editing.
